# Spread of extended-spectrum beta-lactamase producing *Escherichia coli* isolates in Swedish broilers mediated by an incI plasmid carrying *bla*_CTX-M-1_

**DOI:** 10.1186/1751-0147-55-3

**Published:** 2013-01-21

**Authors:** Stefan Börjesson, Björn Bengtsson, Cecilia Jernberg, Stina Englund

**Affiliations:** 1 Department of Animal health and Antimicrobial strategies, National Veterinary Institute (SVA), Uppsala, SE-751 89, Sweden; 2 Department of Preparedness, Swedish Institute for Communicable Disease Control (Nobelsväg 18), Solna, 17182, Sweden

**Keywords:** Extended spectrum betalactamases, ESBL, Broiler, CTX-M-1, *Escherichia coli*

## Abstract

**Background:**

The already high and increasing occurrence of extended-spectrum beta-lactamases (ESBL) producing *Escherichia coli* in European broiler populations is of concern due to the fact that third and fourth generation cephalosporins are deemed critically important in human medicine. In Sweden 34% of the broilers carry ESBL/pAmpC producing *E. coli* in their gut, despite the absence of a known selection pressure such as antimicrobial usages. The aim of the current study was to characterise a selection of *E. coli* strains carrying the *bla*_CTX-M-1_, to determine if the spread was due to a specific clone.

**Findings:**

Ten isolates carrying *bla*_CTX-M-1_ from Swedish broilers belonged to eight different multi-locus sequence types with three isolates belonging to ST155. The ST155 isolates were identical as assessed by PFGE. The *bla*_CTX-M-1_ was in all isolates carried on a plasmid of replicon type incI, which also transferred resistance to tetracycline and sulfamethoxazole.

**Conclusion:**

The occurrence of ESBL-producing *E. coli* in the Swedish broilers is not due to the emergence of a single clone, but rather the spread of a specific incI plasmid carrying *bla*_CTX-M-1_.

## Findings

The increasing trend of extended-spectrum beta-lactamases (ESBL) and plasmid-mediated AmpC (pAmpC) producing *Escherichia coli* in food-producing animals worldwide is worrisome [1,2] due to the significance of third and fourth generation cephalosporins in human medicine. The European Food Safety Authority concluded in a recent report that there exist evidence that food-producing animals can be a reservoir for ESBL and pAmpC producing *Enterobacteriaceae* and the corresponding genes and that transmission has occurred between food-producing animals and humans [3]. The report also highlighted that the high and increasing prevalence in broiler and broiler meat is of particular concern. In addition, a recent study from the Netherlands concluded that identical ESBL producing *E. coli* isolates, plasmids and genes occurred both in broiler, meat and humans [4]. It has been suggested that the reason for the increasing occurrence of ESBL and pAmpC producing *Enterobacteriaceae* in broilers is due to the use of cephalosporins [5]. However, the total sales of third generation cephalosporins devoted to Swedish animals in 2009 constituted ~20 kg, 0.001% of total sales of veterinary antimicrobials, and they were mainly prescribed to horses and cattle [6]. Therefore it was highly surprising when in 2010 34% of broilers in Sweden were found to carry ESBL or pAmpC producing *E. coli* using selective cultivation methods [7]. There were only two genes identified *bla*_CTX-M-1_ and *bla*_CMY-2_, with *bla*_CMY-2_ clearly dominating. This genotype composition differs from that in most other European countries [3]. Other European countries than Sweden generally have a higher diversity of genotypes on their broiler farms and a much lower prevalence of *bla*_CMY-2_, but *bla*_CTX-M-1_ appear to be common in broilers in most countries. A selection of *E. coli* isolates carrying *bla*_CMY-2_ was characterised and compared to human clinical isolates in a previous study [8]. It was shown that the occurrence of *bla*_CMY-2_ was not due to the spread of a single clone and that transmission to humans appears very limited and restricted to the plasmids. In the current study we characterised a selection of *bla*_CTX-M-1_*E. coli* isolates from Swedish broilers and compared the results in a European context.

Ten isolates carrying *bla*_CTX-M-1_ from Swedish broilers (collected February-May 2010) were obtained through the Swedish program for monitoring antimicrobial resistance in the veterinary field (SVARM) [7]. Isolates were obtained by culturing ceacal samples on MacConkey agar with 1 mg/L cefotaxime. All isolates were multi-resistant, i.e. resistant to three or more antimicrobial classes. The isolates were characterised using Pulsed field gel electrophoresis (PFGE) [9], Multilocus sequence typing (MLST) (http://mlst.ucc.ie/) and Polymerase Chain Reaction (PCR) based plasmid replicon typing [10]. For comparison of PFGE pattern DICE coefficient and UPGMA cluster analysis was performed, with position tolerance and optimisation both set at 1%. Transferability of *bla*_CTX-M-1_ was tested through conjugation to *E. coli* HMS174. Transformation was performed on transconjugants PCR positive for multiple plasmid replicon types. Plasmid DNA was extracted according to the protocol by Sambrook and Russell [11] with minor modifications and transformed into ElectroMax™DH10B™ (Gibco Invitrogen, Carlsbad, CA, USA) according to the manufacturer’s instructions, voltage changed to 1250 V. Transconjugants and transformants were tested for antimicrobial susceptibility using VetMIC GN-mo microdilution plates with epidemiological cut-off value set by EUCAST (SVA, Uppsala, Sweden), for ESBL genes [12] and plasmid replicon types.

The 10 isolates belonged to eight different STs, with ST155 (3 isolates) being the most common (Table 1). Nine isolates were typeable using PFGE and 1 was non-typeable. All isolates were able to transfer *bla*_CTX-M-1._ In addition to the ESBL phenotype, all isolates transferred resistance to sulfamethoxazole and tetracycline and one also transferred streptomycin resistance. It was found that the transconjugants contained plasmids belonging to the IncI replicon type. However, five transconjugants were also PCR positive for multiple plasmids replicon types and these five were subjected to transformation. The resulting transformants were all positive for the IncI replicon type and resistant to sulfamethoxazole and tetracycline. Transformation on a transconjugant positive for IncI verified that the IncI plasmid carried *bla*_CTX-M-1_ and sulfamethoxazole and tetracycline resistance phenotype.

**Table 1:** ***Escherichia coli *isolates carrying bla**_***CTX-M-1***_**obtained from broiler chickens characterized by multi locus sequence types (MLST), Pulsed field gel electrophoresis (PFGE) and resistance patterns determined using VetMIC GN-mo microdilution plates**

**MLST (clonal complex)**	**Resistance pattern^A^**	**No. of isolates**
ST57 (CC350)	SuTc	1
ST135 (N.D.^B^)	SuTc	1^C^
ST155 (CC150)	SuTc	3^D^
ST219 (N.D.)	SuTc	1^E^
ST602 (CC446)	SmSuTc	1^C^
ST752 (CC10)	SmSuTc	1
ST1594 (N.D.)	SuTc	1
ST1640 (CC350)	SmSuTc	1

^**A**^Determined in a previous study [7], Su = sulphamethoxazole, Tc = tetracycline, Sm = streptomycin ^B^N.D. = not determined ^**C**^Assigned to the same PFGE-cluster based on a cut-off value of ≥80% ^**D**^Identical pulsed field gel electrophoresis (PFGE) patterns ^E^ Non-typeable by PFGE using *Xba*I.

The results show that the occurrence of ESBL producing *E. coli* in the Swedish broiler population is, like the pAmpC producing *E. coli*[8], not due to one specific clone but appears to be connected to a specific incI plasmid also carrying resistance to sulfamethoxazole and tetracycline. It is well-established that *bla*_CTX-M-1_ on IncI plasmids are frequently isolated both in the poultry industry and in human clinical settings in Europe [2]. In a study from the Netherlands it was also the IncI plasmids carrying *bla*_CTX-M-1_ that dominated and the plasmids were found to be identical in isolates of human and broiler origin [4]. Furthermore, genetically related *bla*_CTX-M-1_-IncI plasmids from human and broiler isolates have also been described in France [13]. It is therefore possible that the Swedish broiler population can be a potential source for these plasmids and genes in human clinical settings. However, to establish if this is the case, further and more extensive studies should be carried out.

Because the incI plasmid carries phenotypical resistance to tetracycline and sulfamethoxazole, usage of these antibiotics, in addition to use of cephalosporins and other B-lactam antibiotics, may facilitate the spread of the plasmid. Previous studies have also suggested that co-resistance has played a role in the rapid emergence of ESBL producing bacteria [2]. However, the occurrence in Sweden of ESBL, nor pAmpC, producing *E. coli* cannot be explained by antibiotic usage because this usage is very limited. For example, in 2011 only 6 of 3185 broiler flocks were treated with antibiotics and no cephalosporins were prescribed [14]. A likely explanation to the high occurrence can be a top-down transmission from imported grand-parent birds. The potential for transmission of cephalosporin-resistant *E. coli*, along with quinolone-resistant isolates, through the production pyramid has been suggested in earlier studies [15,16]. This theory is further supported by a pilot study that identified *E. coli* carrying *bla*_CMY-2_ and/or *bla*_CTX-M-1_ in the environment at production hatcheries and in breeding stocks in Sweden [7]. The high occurrence in Swedish broiler might also be maintained by transmission between flocks. However, the Swedish broiler houses are cleaned and disinfected between batches therefore this type of contribution is probably low.

## Conclusions

The presence of ESBL producing *E. coli* in the Swedish broiler population is not due to the emergence of one specific clone but seems to be due to the spread of one plasmid, an incI plasmid carrying *bla*_CTX-M-1_ and resistance to tetracycline and sulfamethoxazole.

## Competing interests

The authors declare that they have no competing interests.

## Authors’ contributions

SB participated in the design of the study, carried out the analyses and drafted the manuscript. BB participated in the design of the study and assisted and in the drafting of the manuscript. CJ carried out the PFGE-analyses. SE participated in the design of the study helped to draft the manuscript and carried out the analyses. All authors have read, participated in and approved the final manuscript.
